# An introduction to the UK care pathway for children born with a cleft of the lip and/or palate

**DOI:** 10.1038/s41415-023-5998-z

**Published:** 2023-06-23

**Authors:** Tom Dudding, Serena Martin, Sandip Popat

**Affiliations:** 822379796186804587102 Bristol Dental School, University of Bristol, Bristol, UK; The Spires Cleft Centre, John Radcliffe Hospital, Oxford, United Kingdom; 107036866601170321092 The Spires Cleft Centre, John Radcliffe Hospital, Oxford, United Kingdom

## Abstract

Cleft lip and/or palate includes a spectrum of congenital disorders affecting union on the lip, alveolus, hard and/or soft palate. The management of children born with an orofacial cleft requires a complex process from a multidisciplinary team (MDT) to restore form and function. Since the Clinical Standards Advisory Group (CSAG) report in 1998, the UK has reformed and restructured cleft services to improve the outcomes for children born with a cleft.

The spectrum of cleft conditions, the members of the MDT and a chronological description of the stages of cleft management from diagnosis to adulthood are described using a clinical example. This paper forms the introduction to a series of more detailed papers which span all major aspects of cleft management. The papers will cover the following topics: dental anomalies; associated medical conditions among children; orthodontic management of patients; speech assessment and intervention; role of the clinical psychologist; challenges for the paediatric dentist; genetics and orofacial clefts; surgery - primary and secondary; restorative dentistry; and global perspectives.

## Key points

- Presents an introduction to cleft lip and/or palate.
- Provides an introduction to the multidisciplinary cleft team.
- Gives an overview of stages for cleft management from diagnosis to adulthood.

## Introduction to cleft lip and plate

Cleft lip and/or palate includes a wide spectrum of congenital disorders which affect the union of the lip, alveolus, hard palate and/or soft palate. A cleft lip can be either unilateral or bilateral with varying degrees of completeness, with some children also having a cleft of the alveolus and palate. An isolated cleft of the palate is distinct from a cleft of the lip and palate and can involve only the soft palate or include the hard and soft palate up to the incisive foramen. The mildest subtype of cleft palate is known as a submucous cleft palate, where the underlying muscular anatomy is deficient in the midline; however, the mucosa remains intact and can be termed a 'hidden cleft'.^1^

**Fig. 1:**
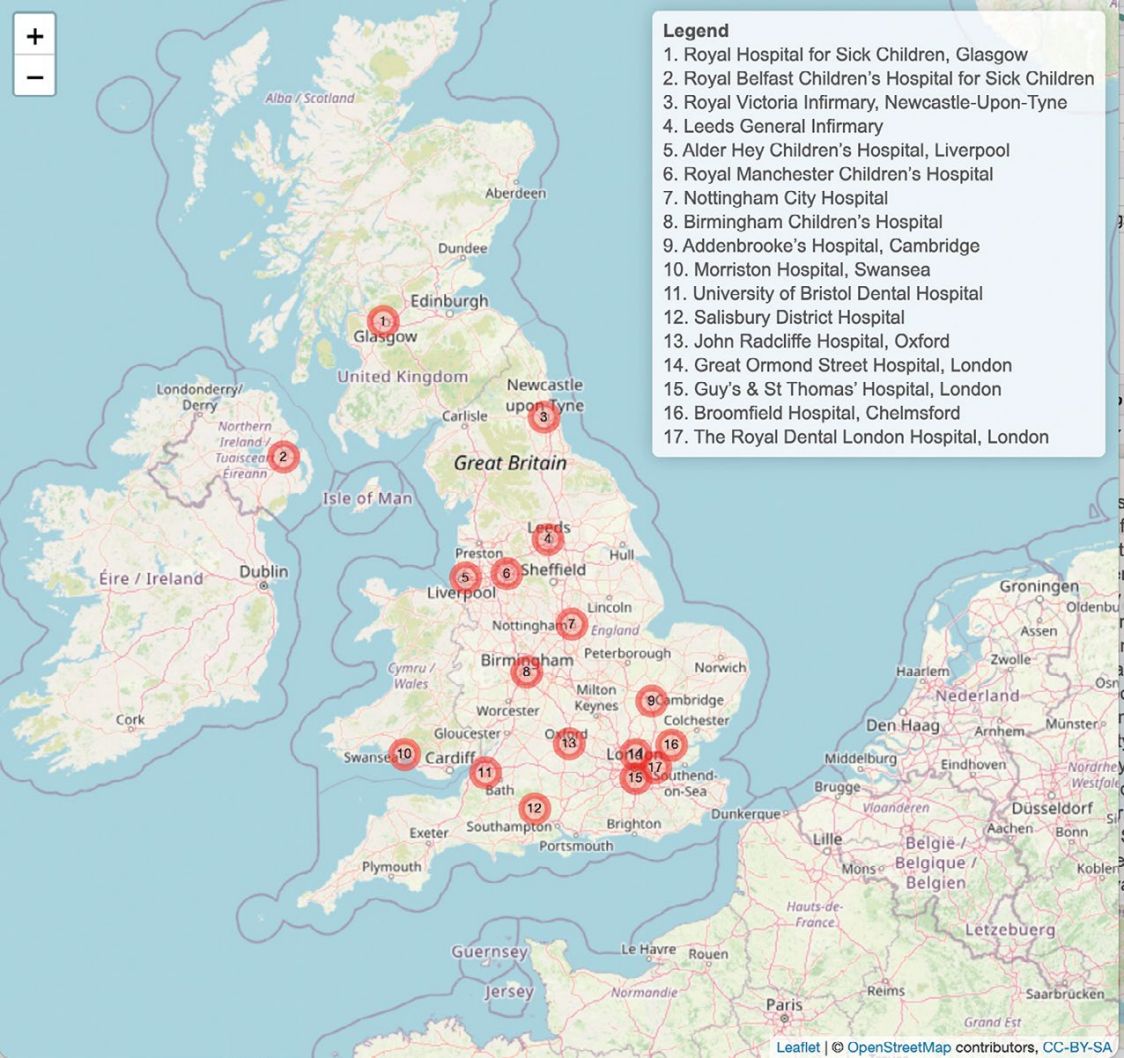
Map of cleft centres. Map created in leafletR. Data from OpenStreetMap under the Open Database Licence

The management objectives for children born with an orofacial cleft are to restore both form and function, with reconstruction of the underlying anatomy being required to ensure normal function of both the lip and palate. The outcomes are achieved by input from a wide range of associated specialities which together make up the cleft multidisciplinary team (MDT). Prior to the Clinical Standards Advisory Group (CSAG) report in 1998, there were 57 UK centres treating cleft patients; treatment was inconsistent and clinical outcomes were poor.^1^^,^^2^ The 15-month CSAG study was accepted by the UK government in 1998 and recommended 8-15 multidisciplinary cleft centres nationally, in a process often referred to as 'centralisation'. Three years later, nine cleft services were set up. Within each service, there may be more than one cleft unit (or centre) staffed by the cleft MDTs (Fig. 1).

This series of papers aims to provide a dentally relevant understanding and overview of the management of children born with an orofacial cleft in the UK. This first paper outlines the journey that a child born with a cleft will follow throughout their life, from antenatal diagnosis *in utero* to adulthood. The majority of interactions with the cleft MDT occur early in the child's life; as the child matures, the routine planned appointments reduce in frequency, unless there are any issues requiring active management and input from any of the MDT members.

The papers that follow in this series will focus on various aspects of the MDT and provide an overview of the management focus and input required by each subspeciality to obtain the best outcome for these children.

**Fig. 2:**
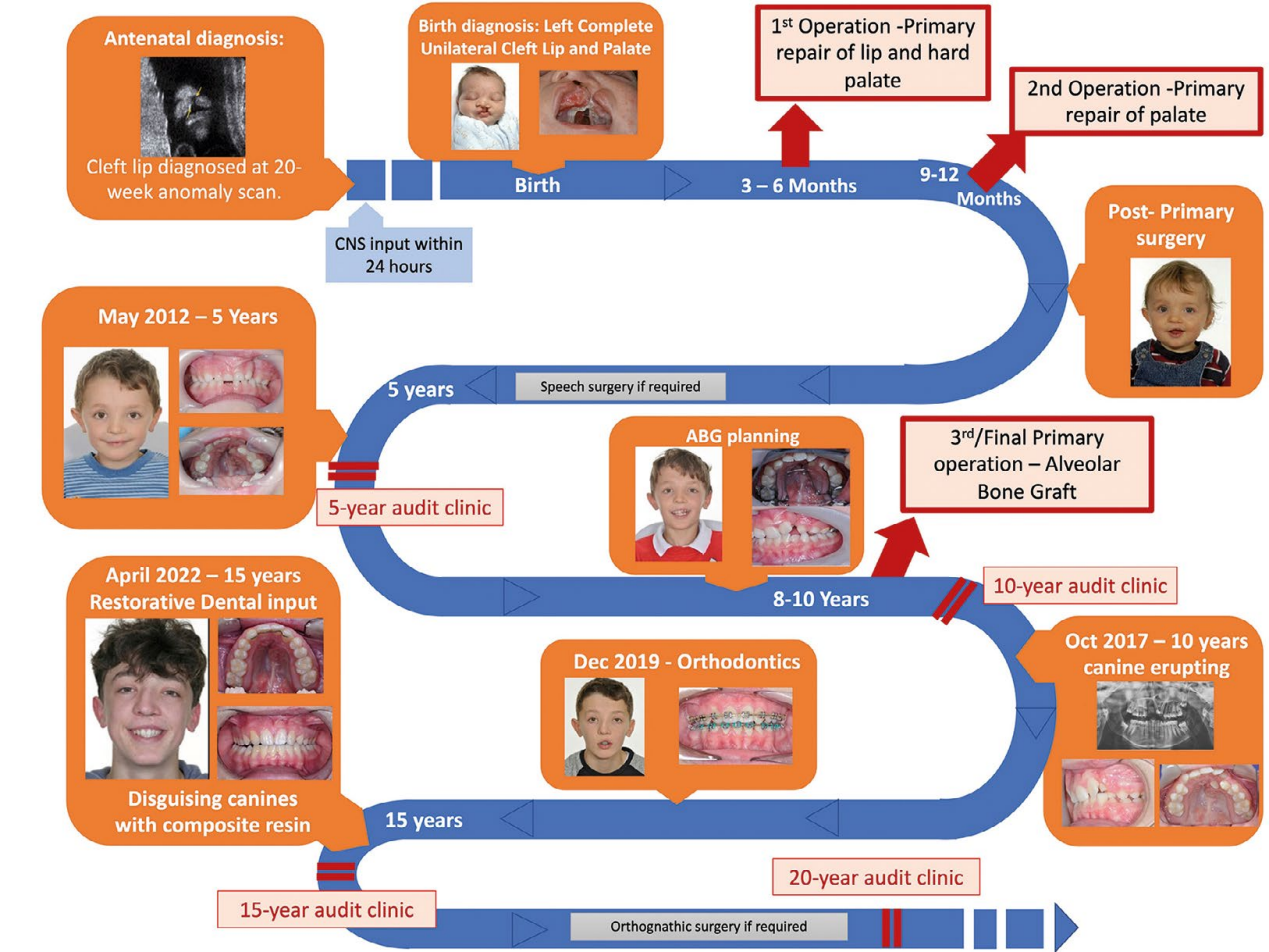
Infographic detailing the clinical journey of one UCLP patient. Although this journey is typical of a UCLP patient, each patient journey will differ depending on their clinical presentations. The journey will differ for different cleft types, for example, an isolated cleft palate patient will not need lip repair or ABG and is less likely to need complex orthodontics and tooth replacement

Figure 2 provides a step-by-step overview of the cleft MDT pathway using a real patient born with a left-sided unilateral cleft lip and palate (UCLP). This case study will focus specifically on the management of a patient born with a complete UCLP. Although the pathway remains similar for patients born with other subtypes of orofacial clefts, the exact management of other patients can be gleemed in more detail by reading the relevant papers in this series.

## The patient journey

### Antenatal diagnosis

Approximately 81% of babies with a cleft lip are diagnosed antenatally.^3^ Detection of a cleft lip is one of the 11 conditions specifically checked for during the NHS foetal anomaly scan between 18-21 weeks of gestation. As this scan produces a 2D image, it is virtually impossible to determine if the palate is intact, and some centres will offer a magnetic resonance imaging scan to assess the palate in more detail. Following an antenatal diagnosis, the cleft clinical nurse specialist (CNS) will make contact with the parent(s) within 24 hours and will provide much needed support and counselling in preparation for the birth of their baby. They will remain as a key contact for the parent(s) throughout the remainder of the pregnancy and until the birth of the baby and beyond.

### Birth and early months

Following birth, the CNS will visit the family within 24 hours, which may be while they are still in hospital or following discharge home. The CNS has a key role in supporting parents, particularly with feeding in the early days, which is considered to be one of the major concerns for parents.^4^ The presence of a cleft affecting both the lip and palate means the baby is unable to generate sufficient oral pressure to enable suction from either a nipple or the teat of a bottle. Commonly, special feedings bottles are required, including bottles which allow the parent(s) to 'squeeze' and simulate the suction for the baby. This requires training to ensure the 'squeeze' is timed simultaneously with the baby attempting to suck.

### Lip repair: 3-6 months

The parent(s) and the baby will meet the cleft surgeon, along with other members of the MDT, during the first outpatient appointment. For a child with a UCLP, the first surgical procedure is the lip repair, which is typically completed at between the age of 3-6 months. The hard palate will also be repaired at this time, but the alveolar cleft is typically left until a later age to preserve facial growth.^5^^,^^6^

### Palate repair: 9 months to 1 year

The remainder of the palate will be repaired between 9-13 months of age. The aim of the palate repair is to provide an intact palate with normal velopharyngeal function to enable speech development.^7^

### 1-5 years of age

These early years following palate repair are critical for speech development and the child will be monitored closely by the cleft team, particularly the cleft specialist speech therapists. At 18 months, and again at three years, the child will have a speech assessment; the exact timing will depend on the number of words the child has developed to ensure an adequate speech sample can be obtained. The aim is to ensure the child has normal speech before starting school. In approximately one-third of patients, a second procedure may be required to improve speech.^8^

Access to advice from a paediatric dentist is available as the deciduous teeth begin to erupt. The role of the general dentist also begins at this early stage and the importance of dental hygiene cannot be overstated. Patients with a cleft have a higher rate of dental caries (decayed, missing, filled teeth = 0.63 [95% CI: 0.46, 0.79]; higher in individuals with cleft lip and palate compared to those without)^9^ and other abnormalities, including hypodontia (up to 70%)^10^ and enamel hypoplasia, which may require treatment.^11^

At the age of five, the child will attend the cleft unit for the first of up to four audit clinics. At this visit, the child is assessed by dental and orthodontic teams, audiology, psychology, clinical photography, and speech and language therapy. The primary aim of the visit is to identify any arising problems but also to record a series of metrics for the national Cleft Registry and Audit NEtwork (CRANE) database (see CRANE section below).

### 6-12 years of age

As the child's permanent dentition starts to erupt, this period is important for their dentoalveolar development. Children with a complete cleft lip and palate need to be assessed by the cleft surgeon, orthodontist and dentist to start planning their alveolar bone graft (ABG). A panoramic radiograph (orthopantomagram) and an upper occlusal x-ray are both taken to assess the alveolar cleft, as well as the dental development and the presence of any supernumerary teeth (present in to to 35% of cases) that may require removal pre-operatively.^7^A decision is also made at this time if any orthodontic work is required to enable access to the alveolar cleft, for example, arch expansion. The ABG will stabilise the anterior maxilla and provide bony support for the permanent dentition, as well as for future orthodontic appliances. In the UK, the timing of the ABG is based on dental development, with the aim of performing the bone graft before the permanent canine erupts.^6^

**Fig. 3:**
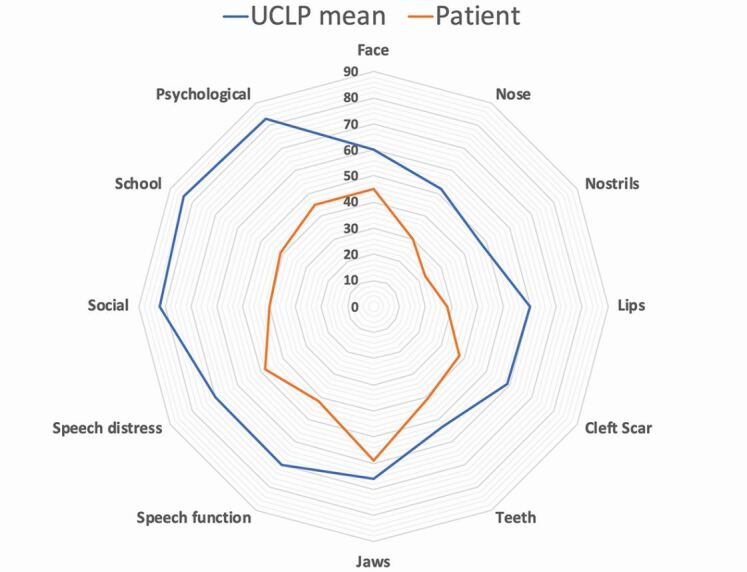
Example radar plot showing patient reported outcome measures (orange line) and mean UCLP phenotype (blue line). The patient has scores which fall below the mean for the cleft phenotype for all parameters

The second of the four audit clinics occurs at age 10 and follows the same format as the age five audit clinic. By this stage, the ABG should have been performed. Several cleft centres make use of patient-reported outcome measures (PROMs). One example is the CLEFT-Q questionnaire^9^ which allocates a patient-reported score to aspects of appearance, health and quality of life. The results of these PROMs can be compared to means for that cleft phenotype and thus tailor the clinical consultation. An example of a radar plot is shown in Figure 3; note a high score represents a good outcome.

### 12-20 years

The majority of cleft patients will require orthodontics in adolescence, which may include exposing and bonding unerupted canines to assist with their eruption into the ABG site. Specialist restorative dental input may also be required for replacement of missing teeth or reshaping of misshapen teeth, for example, disguising canines as lateral incisors.

The third audit clinic occurs at age 15. It is designed to identify any issues that may need to be managed as the patient is nearing the end of growth. This may include the need for orthognathic surgery, or if there are any functional or aesthetic concerns which necessitate surgical revision of the lip, or a cleft septorhinoplasty.^12^

Patients with UCLP may develop a Class III skeletal relationship. This is thought to be due to a combination of maxillary hypoplasia secondary to the cleft itself, as well as the impact of surgical repair and scarring during growth. If this is mild, it may be managed by orthodontics alone; however, some patients with a more severe occlusal abnormality may require orthognathic surgery. This may involve advancement of the maxilla alone, or in those with larger discrepancies, a mandibular setback may also be required to align the top and bottom jaws. As with all major decisions along the pathway, the psychologist will work closely with the patient to assist in the decision-making process.

A final audit clinic is conducted around the age of 20 years. If there are no ongoing issues or concerns that require active input from the cleft team, then the patient can be discharged from the cleft service at this point.

### Adulthood

After being discharged from the cleft pathway, adults may return to any of the cleft MDT teams at any stage if any concerns arise. This may focus on specialist restorative dental care^13^ as previous treatments begin to fail, or it may involve genetic counselling as the patient begins to consider having their own family.

## CRANE

CRANE is a database, set up in 2000, which collects information about children born with a cleft lip and palate in England, Wales and Northern Ireland. Scotland has also recently started submitting data to CRANE. It has two core aims: 1) to collect birth, demographic and epidemiological information on all children born in England, Wales and Northern Ireland with a cleft lip and/or palate; and 2) to collect information on the treatment of children with a cleft lip and/or palate and the outcome of these interventions. The data recorded in the database is collected by the local cleft teams during treatment and from five-yearly audits of each patient. CRANE produces annual reports and is involved in research, commissioning services with the aim of improving the care of patients with cleft.

The CRANE outlier policy facilitates monitoring of each cleft centre's performance indicators.^14^ Key performance indicators, such as timing of palate repair (by 13 months), are compared against established benchmarks and outcomes, and are compared against the national average. The policy aims to identify situations where centres are between two and three standard deviations below the national average (flagged as an 'alert') or more than three standard deviations below the national average (flagged as 'alarm'). Where outliers are identified, there are robust processes for investigation and quality improvement to ensure services are improved. This remains true for units that are identified as positive outliers where key learning points can be gleaned from their success.

## Cleft research studies

The UK hosts the largest prospective longitudinal cohort of cleft patients. The Cleft Collective, which continues to recruit, involves over 3,500 children with orofacial clefts and includes biological samples, and phenotypic, clinical and genetic data (http://www.bristol.ac.uk/dental/cleft-collective/). Including family members, the study has collected data on over 10,000 participants. Another study, Cleft Care UK, which is closed for recruitment, was a cross-sectional study conducted in 2015, which aimed to investigate the effects of centralisation 15 years after the CSAG report. This study reported its finding across different outcomes,^15^^,^^16^^,^^17^^,^^18^^,^^19^^,^^20^showing an overall improvement in surgical, occlusal, speech and language, facial proportions and psychological outcomes, but no improvement in dental and hearing outcomes.

## Summary

Patients born with cleft lip and palate are managed by an MDT approach that involves a wide range of health care professionals. Each patient journey is unique but there are phases common to many patients with the same cleft phenotype. The burden of care for both patients and their families is high, but the treatment outcomes can be lifechanging. Dentistry is key to many aspects of cleft care, and specialist and general dentists alike will often be involved with the ongoing management of cleft patients throughout their professional career.
